# The Association Between Diabetes Mellitus and Osteoarthritis: Does Diabetes Mellitus Play a Role in the Severity of Pain in Osteoarthritis?

**DOI:** 10.7759/cureus.21449

**Published:** 2022-01-20

**Authors:** Tutul Chowdhury, Amulya Bellamkonda, Nicole Gousy, Padmaja Deb Roy

**Affiliations:** 1 Internal Medicine, One Brooklyn Health System, Brooklyn, USA; 2 Medicine, American University of Antigua, New York, USA; 3 Department of Medicine, Comilla Medical College, New York, USA

**Keywords:** obesity, metabolic syndrome and osteoarthritis, articular cartilage, chronic joint pain, osteoarthritis (oa), diabetes mellitis

## Abstract

Osteoarthritis (OA) is one of the most prevalent degenerative joint diseases, which results in the inevitable destruction of joints leading to pain and joint immobility. Some studies have reported a potential link between diabetes mellitus (DM) and the worsening symptoms and severity of OA. Based on our literature review, the microcellular environment of patients with DM showed accelerated joint destruction and increased inflammation in every anatomical aspect of the joint including the bones, tendons, ligaments, cartilage, and synovium. Additionally, the biomechanical and biochemical properties of these tissues were more severely impacted in patients with DM and OA compared to those without DM, suggesting that DM plays an important role in the pathogenesis of OA. Specifically, we found that advanced glycation end products (AGEs) are the key to inducing the acceleration of joint destruction; however, their role in the pathogenesis has yet to be fully mapped out. In this narrative review, we aim to explore the role that DM plays in the acceleration of OA leading to increased reports of joint pain in those with both diseases. We believe this topic of discussion to be important due to the increased prevalence of both diseases over the last several decades, potentially leading to an increased medical burden on both patients and the community at large.

## Introduction and background

Osteoarthritis (OA) is one of the most prevalent joint disorders, and it leads to diarthrodial articular cartilage deterioration with eventual disability in adults [1,2]. The prevalence, progression, and severity of the symptoms of OA can be affected by multiple factors such as comorbidities, lifestyle, diet, age, and genetics [3-5]. Recent studies have suggested phenotypically subcategorizing OA to better understand the pathogenesis and causes related to OA. These subtypes entail age-related, post-traumatic event-related, and metabolic syndrome-related categories [1]. By breaking OA down into categories, we can further evaluate how other preexisting conditions and differences in lifestyle can affect the progression of OA. This issue is especially relevant given the rise in the prevalence of metabolic syndrome-related OA [1]. The comorbidities and risk factors in OA pathogenesis share common features with those seen in type 2 diabetes mellitus (DM) [1]. Since the 1980s, it has been observed that those diagnosed with OA also have a preexisting DM diagnosis, and vice versa. Owing to their frequent coexistence, OA and DM have a substantial impact on the health burden on both the individual and the wider community [4,5]. The US Third National Health and Nutrition Examination Survey (NHANES III) recently performed a cross-sectional study, which demonstrated a higher prevalence of DM in those with OA as compared to the general population [4]. Furthermore, it was reported that DM plays a unique role of its own in the progression and pathogenesis of OA [4,6]. Even hyperglycemia alone was reported to be associated with a separate mechanism on the pathogenesis and advancement of OA through the accumulation of advanced glycation end products (AGEs), oxidative stress, and dysregulation of articular cartilage metabolism [1,2].

OA is a degenerative disease leading to the inevitable destruction and disfigurement of joints in adults. This condition occurs with the common symptoms of pain and joint immobility, which can be the initial motivation for patients to consult a physician for relief. Pain in OA is one of the most reported symptoms and ranges drastically in severity on a multifactorial basis, with one of the etiologies appearing to branch from DM. In a study where patients were assessed using the Knee injury and Osteoarthritis Outcome Score (KOOS) and the Western Ontario and McMaster Universities Arthritis Index (WOMAC), those with coexisting DM and OA more frequently reported more severe symptoms compared to those without [1,7]. Those with poorer glycemic control reported an increased pain severity compared to those with DM with a better controlled HbA1C level [3]. Using the WOMAC Index, one study revealed that patients diagnosed with DM and OA reported significantly increased severity and frequency in pain flare-ups, increased incidences of bilateral synovitis, and increased likelihood for requiring knee arthroplasty compared to those with OA alone [7]. In a 20-year longitudinal study predicting the outcome of patients with OA and DM independent of obesity and age, those with DM were more likely to receive knee and/or hip joint replacement surgery than nondiabetic patients (p=0.006) [8]. Additionally, they reported bilateral synovitis to be significantly more common in diabetics than nondiabetics (p=0.004) [8].

In this narrative review, we aim to explore the relationship between DM and the progression of OA in terms of patient-reported pain severity. We intend to review the related literature and report how elevated glucose levels in DM play a role in the pathogenesis of OA from the anatomical level to the metabolic level. Based on the literature review, we report on DM influencing the musculoskeletal system, specifically the bones, tendons, ligaments, and cartilage of joints most frequently affected in OA. We will further discuss how DM interferes with the mechanism and mediators involved with joint destruction. The goal of this review is to shed light on the relationship between these two incredibly common diseases to help reduce the severity of pain experienced by patients with OA.

Methodology

**Figure 1:**
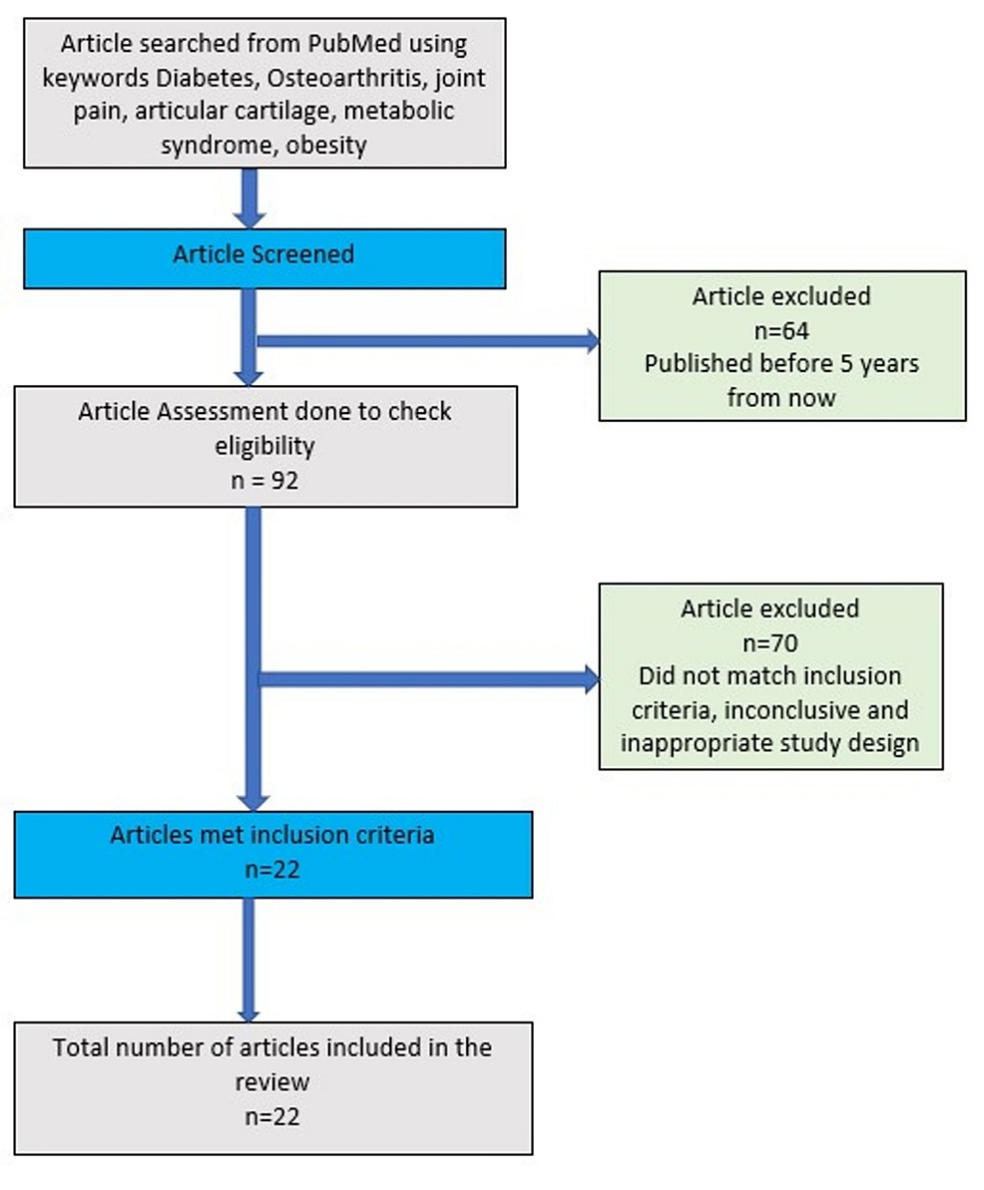
Flowchart of the search strategy and screening process

Data collection was performed, without any language restriction, using the databases PubMed/Medline, BIREME, and Cochrane, as well as the references found within these articles. Three reviewers performed independent data extractions of the articles chosen for this narrative review. Subsequently, these reviewers further scrutinized the articles to ensure their methodological quality (Figure 1).

## Review

Impact of diabetes on the musculoskeletal system, bone, and articular cartilage

**Figure 2:**
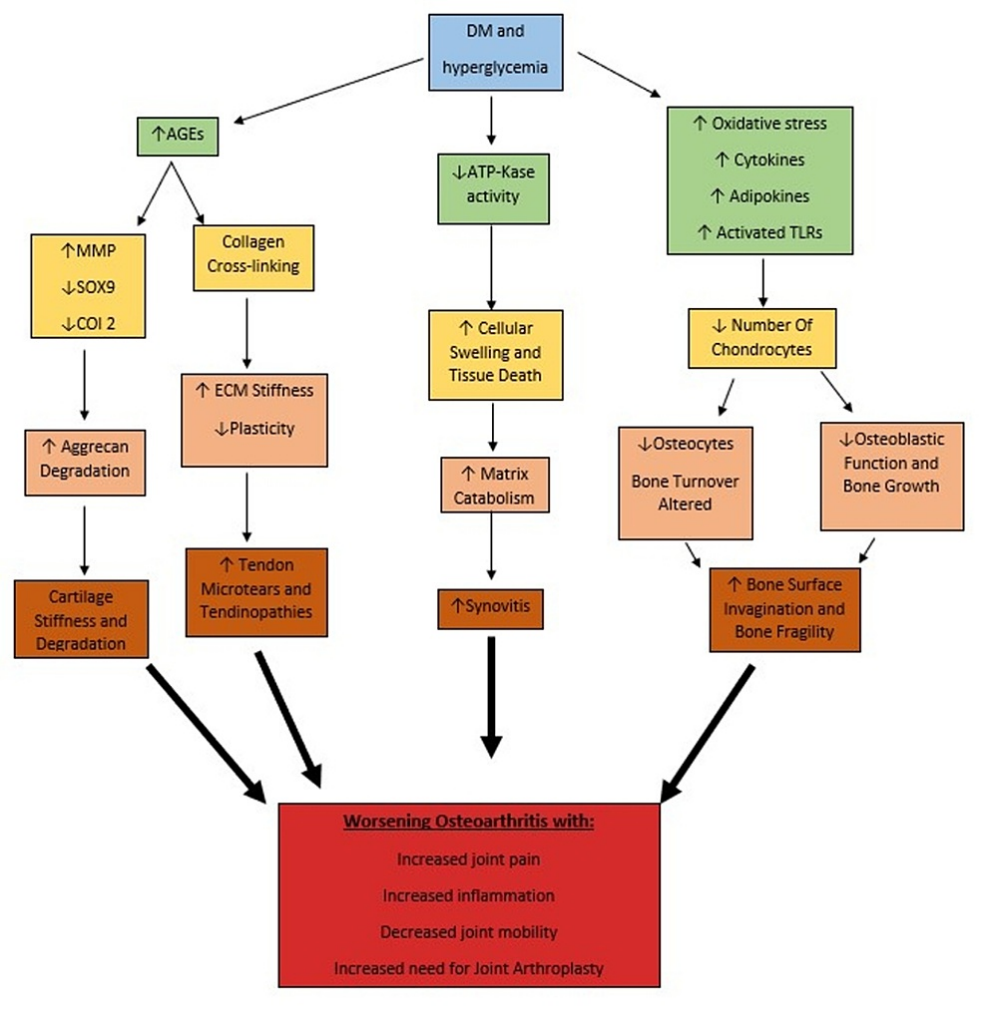
Flowchart showing the effects of hyperglycemia on the progression of osteoarthritis DM: diabetes mellitus; AGEs: advanced glycation end products; ATPKase: ATP-K+ channel; TLRs: toll-like-receptors; SOX9 and COI 2: chondrocytes-specific proteins; MMP: matrix metalloproteinase; ECM: extracellular matrix

Metabolism of bone and articular cartilage can be greatly affected by a hyperglycemic condition, which could prove to be detrimental [1]. The integrity of articular cartilage and extracellular matrix can become compromised in a proinflammatory environment; it is a given that diabetic patients are more prone to have inflammatory changes in the cellular environment [2,3]. Studies performed on diabetic mice have exhibited increased loosened and tangentially arranged collagen fiber, moderate to severe loss of chondrocytes, and increased bone surface invagination in contrast to healthy mice sharing other common risk factors [4]. The histological severity of OA was also remarkably higher in the diabetic group compared to the control group of mice. A diabetic microenvironment facilitates the development of OA by promoting accelerated synthesis and accumulation of AGEs, further increasing the oxidative burden by impairing healing and elasticity [6] (Figure 2). A study conducted by Dubey et al. concluded that the increased glycation products in diabetic mice increasingly induce matrix metalloproteinase expression as well as reduce chondrocytes-specific protein expressions such as SOX9, COl 2, and aggrecan [3]. In human studies, altered mineral properties, deleterious tissue changes, and excessive compressive stiffness have been observed in a diabetic experimental group. This suggests that DM can further complicate osteoarthritic joint pathology [3,7].

Impact of diabetes on tendons and ligaments

Hyperglycemia can lead to biomechanical and biochemical tendon abnormalities, which contribute to debilitating tendinopathies, further exacerbating OA. Several studies have consistently shown that hyperglycemic states lead to increased AGE levels that have detrimental effects on the integrity of the chemical structure of collagen resulting in increased microtears [9,10]. Additionally, AGEs seem to have an inhibitory impact on tendon-derived stem/progenitor cells (TSPCs), which can decrease their ability to maintain tendon homeostasis and reduce the risk of tendinopathy development [9-11]. These changes contribute to the decreased rupture threshold that can exacerbate OA in patients with DM [11].

It is very well known that collagen has a triple helix structure, which contributes to collagen’s inherent strength, stability, and linear organization within the tissues of tendons ligaments and cartilage [9,12]. AGEs aggregate on all subtypes of collagen but specifically type I collagen most frequently due to its increased affinity for AGEs and its increased availability within the tendon extracellular matrix [9,13]. Studies have shown that the arrangement of collagen in tendons in those with DM is significantly more disorganized due to AGE cross-linking between collagen fibers, disrupting the normal triple helix [10,12]. This cross-linking results in significant stiffening of the extracellular matrix, reducing its plasticity and ability for tendon-fiber sliding [9-13]. Since type I collagen has a half-life of roughly one to two years, there is an increased opportunity for collagen cross-linking in those with DM, leading to an exponentially increased risk for tendinopathies that can further exacerbate OA [9,14].

Along with interfering with the mechanical structure of tendons, AGEs also seem to play a role in increasing TSPC apoptosis, decreasing their ability to maintain tendon homeostasis and repair of extracellular matrix [9,10]. While the relationship between AGEs and TSPCs is still unclear, it is apparent that they are connected in the pathogenesis of tendinopathies in DM [10]. Further investigation of the effects of AGEs on TSPCs, collagen, and tendons and ligaments can help reduce tendon pain that often accompanies OA and DM. Since exercise is a keystone in the management of DM, understanding how tendons are affected in DM can reduce overuse injuries that can interfere with disease-modifying exercise.

Mechanism and mediators involved in joint destruction

Effects of DM on joints are complex; King et al. have clearly explained the effects of diabetes and hyperglycemia on bone, articular cartilage, synovium, tendons, and ligaments [11]. In T1DM, insulin deficiency impairs osteoblastic bone formation and inhibits bone mass during growth; additionally, AGEs may, directly and indirectly, alter matrix properties [4,15,16]. Whereas in T2DM, a constellation of factors such as hyperglycemia, inflammatory cytokines and adipokines, oxidative and osmotic stress, and AGEs collectively inhibit osteocyte function, alter bone turnover, and degrade collagen properties [4,16]. Significant synovitis occurring in OA may be exacerbated by the increased levels of inflammatory cytokines, adipokines, and prostaglandins seen in DM tissues [17]. Signaling through pathways of innate immunity, such as toll-like receptors, may also produce inflammation in both DM and OA [15].

**Figure 3:**
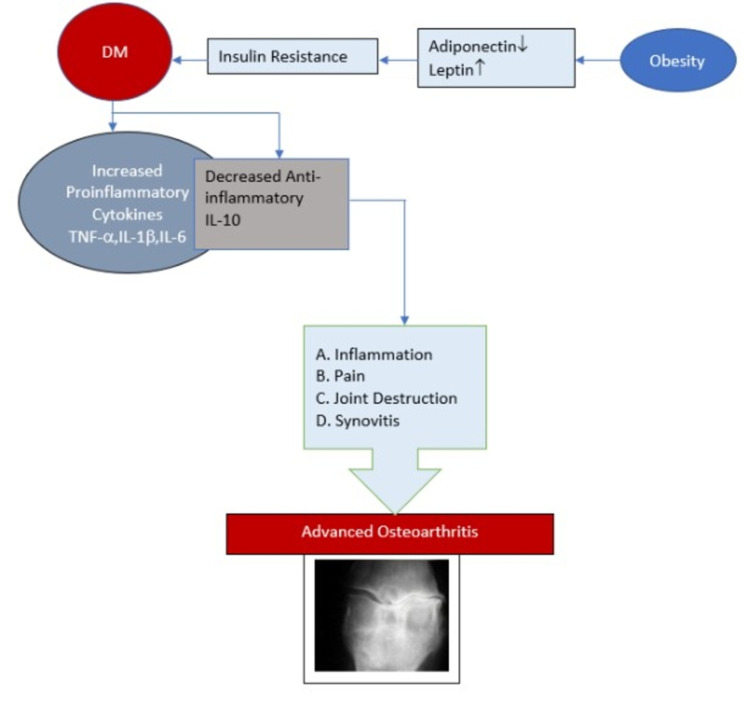
Schematic presentation of the interrelation of diabetes and osteoarthritis regarding joint destruction DM: diabetes mellitus; TNF: tumor necrosis factor; IL: interleukin

A hyperglycemic environment increases the production of reactive oxygen species and oxidants and promotes matrix catabolism. In these environments, cellular transport of glucose becomes critical, which, if altered as in DM, contributes to excess oxidative stress and tissue damage and accelerates OA; this was extensively explained by Rosa et al. in two different studies (Figure 3) [18,19]. The effects of high glucose may be associated with the impaired function of ATP-sensitive K+ channels, which couples GLUT channels to intracellular ATP/ADP levels and membrane potential [20].

High tissue levels of glucose also accelerate the formation of AGEs. The AGEs signal through RAGE (receptor for AGEs) and other receptors and produce various deleterious effects on chondrocytes such as inflammation and cytokine-mediated catabolism. Also, cross-linking of collagen by AGEs alters tissue biomechanical properties [21] and may also inhibit extracellular matrix turnover by restricting access to proteolytic sites [22]. However, Vos et al. clearly stated in their hypothesis that elevated cartilage AGEs do not accelerate the development of early canine OA upon minimal surgical damage [23]. Thus, whether or how AGEs play a significant role in OA remains unclear.

## Conclusions

Since the prevalence of both OA and DM is increasing, the relationship between these two diseases needs to be investigated to help reduce the health burden on both the individual and the community of those affected. It can be concluded that DM has a crucial role in the acceleration of OA pathogenesis even in its early onset; however, the definitive causal pathway still needs to be established. Based on the available literature, chronic hyperglycemic states can increase inflammation in the microcellular environments of joints. Although the complete pathogenesis is not mapped out, AGEs have a central role in perpetuating inflammatory processes in every anatomical part of the joint. This increased inflammation can then exponentiate the breakdown of cells and tissues, potentiating the progression of OA. Therefore, an individual with poor glycemic control is more prone to have osteoarthritic pain with more severity in contrast to one with OA only. Choosing appropriate anti-diabetic agents to decelerate the rate of subchondral bone changes in OA could be an excellent topic for further studies. Specifically, further investigation on how AGEs exactly affect chondrocytes through the RAGE receptor should be pursued as decreased chondrocyte function correlates to increased pathological fractures and increased need for joint arthroplasty. Furthermore, early lifestyle changes, dietary modification, and intensive glycemic control should be practiced and encouraged to delay the onset and severity of the pain in OA.

## References

[1] Zaharia OP, Pesta DH, Bobrov P (2021). Reduced muscle strength is associated with insulin resistance in type 2 diabetes patients with osteoarthritis. J Clin Endocrinol Metab.

[2] Rehling T, Bjørkman AD, Andersen MB, Ekholm O, Molsted S (2019). Diabetes is associated with musculoskeletal pain, osteoarthritis, osteoporosis, and rheumatoid arthritis. J Diabetes Res.

[3] Dubey NK, Ningrum DN, Dubey R (2018). Correlation between diabetes mellitus and knee osteoarthritis: a dry-to-wet lab approach. Int J Mol Sci.

[4] Lekkala S, Taylor EA, Hunt HB, Donnelly E (2019). Effects of diabetes on bone material properties. Curr Osteoporos Rep.

[5] Kharroubi AT, Darwish HM (2015). Diabetes mellitus: the epidemic of the century. World J Diabetes.

[6] Wang HJ, Giambini H, Chen JW (2021). Diabetes mellitus accelerates the progression of osteoarthritis in streptozotocin-induced diabetic mice by deteriorating bone microarchitecture, bone mineral composition, and bone strength of subchondral bone. Ann Transl Med.

[7] Piva SR, Susko AM, Khoja SS, Josbeno DA, Fitzgerald GK, Toledo FG (2015). Links between osteoarthritis and diabetes: implications for management from a physical activity perspective. Clin Geriatr Med.

[8] Schett G, Kleyer A, Perricone C (2013). Diabetes is an independent predictor for severe osteoarthritis: results from a longitudinal cohort study. Diabetes Care.

[9] de Jonge S, Rozenberg R, Vieyra B (2015). Achilles tendons in people with type 2 diabetes show mildly compromised structure: an ultrasound tissue characterisation study. Br J Sports Med.

[10] Shi L, Lu PP, Dai GC, Li YJ, Rui YF (2021). Advanced glycation end productions and tendon stem/progenitor cells in pathogenesis of diabetic tendinopathy. World J Stem Cells.

[11] King KB, Rosenthal AK (2015). The adverse effects of diabetes on osteoarthritis: update on clinical evidence and molecular mechanisms. Osteoarthritis Cartilage.

[12] Gautieri A, Passini FS, Silván U (2017). Advanced glycation end-products: mechanics of aged collagen from molecule to tissue. Matrix Biol.

[13] Ahmed N (2005). Advanced glycation endproducts--role in pathology of diabetic complications. Diabetes Res Clin Pract.

[14] Verzijl N, DeGroot J, Thorpe SR (2000). Effect of collagen turnover on the accumulation of advanced glycation end products. J Biol Chem.

[15] Rajamani U, Jialal I (2014). Hyperglycemia induces Toll-like receptor-2 and -4 expression and activity in human microvascular retinal endothelial cells: implications for diabetic retinopathy. J Diabetes Res.

[16] Hough FS, Pierroz DD, Cooper C, Ferrari SL (2016). Mechanisms in endocrinology: mechanisms and evaluation of bone fragility in type 1 diabetes mellitus. Eur J Endocrinol.

[17] Stenholm S, Koster A, Alley DE (2010). Adipocytokines and the metabolic syndrome among older persons with and without obesity: the InCHIANTI study. Clin Endocrinol (Oxf).

[18] Rosa SC, Rufino AT, Judas F, Tenreiro C, Lopes MC, Mendes AF (2011). Expression and function of the insulin receptor in normal and osteoarthritic human chondrocytes: modulation of anabolic gene expression, glucose transport and GLUT-1 content by insulin. Osteoarthritis Cartilage.

[19] Rosa SC, Gonçalves J, Judas F, Mobasheri A, Lopes C, Mendes AF (2009). Impaired glucose transporter-1 degradation and increased glucose transport and oxidative stress in response to high glucose in chondrocytes from osteoarthritic versus normal human cartilage. Arthritis Res Ther.

[20] Hart DJ, Doyle DV, Spector TD (1995). Association between metabolic factors and knee osteoarthritis in women: the Chingford Study. J Rheumatol.

[21] Li Y, Fessel G, Georgiadis M, Snedeker JG (2013). Advanced glycation end-products diminish tendon collagen fiber sliding. Matrix Biol.

[22] DeGroot J, Verzijl N, Jacobs KM (2001). Accumulation of advanced glycation endproducts reduces chondrocyte-mediated extracellular matrix turnover in human articular cartilage. Osteoarthritis Cartilage.

[23] Vos PA, DeGroot J, Barten-van Rijbroek AD, Zuurmond AM, Bijlsma JW, Mastbergen SC, Lafeber FP (2012). Elevation of cartilage AGEs does not accelerate initiation of canine experimental osteoarthritis upon mild surgical damage. J Orthop Res.

